# Predictors and Causes of Intraoperative Conversion From Off-Pump to On-Pump Coronary Artery Bypass: A Retrospective Study

**DOI:** 10.7759/cureus.73177

**Published:** 2024-11-06

**Authors:** Waqar Masud Malik, Kifayat Ullah, Muhammad Ali Gohar, Sobia Siddique, Hira Hameed, Muhammad Mansoor Tariq, Amir Iqbal, Muhammad Nisar, Ubaid Ur Rahman, Muhammad Abdul Haseeb, Fahad R Khan

**Affiliations:** 1 Cardiac Surgery, Peshawar Institute of Cardiology, Peshawar, PAK; 2 Cardiac Surgery, Armed Forces Institute of Cardiology, Rawalpindi, PAK; 3 Cardiac Surgery, Peshawar institute of Cardiology, Peshawar, PAK; 4 Interventional Cardiology, Peshawar Institute of Cardiology, Peshawar, PAK; 5 General Cardiology, Lady Reading Hospital, Peshawar, PAK

**Keywords:** coronary artery bypass, hemodynamic instability, intraoperative conversion, left main stenosis, off-pump coronary artery bypass, oncab, on-pump coronary artery bypass, opcab

## Abstract

Objective: This study evaluated the incidence, causes, and predictors of intraoperative conversion from off-pump coronary artery bypass grafting (OPCAB) to on-pump coronary artery bypass grafting (ONCAB), identifying risk factors to improve surgical planning.

Methods: This retrospective case-control study included patients who underwent OPCAB at the Peshawar Institute of Cardiology, Peshawar, Pakistan, between December 8, 2021, and December 7, 2023. Among 714 patients, 27 (3.78%) required conversion to ONCAB. For comparison, 108 (15.1%) controls were randomly selected from those who completed OPCAB successfully. Preoperative and intraoperative data were analyzed, and logistic regression identified predictors of conversion.

Results: The most frequent cause of conversion was hemodynamic instability, which occurred in 18 (66.67%) cases. Persistent hypotension and ST-segment changes lasting approximately 10-20 minutes were primary indicators before conversion, highlighting the need for close intraoperative monitoring. Left main coronary artery stenosis of 50%-70% emerged as the only independent predictor of conversion (odds ratio (OR) 7.60, 95% CI: 2.91-19.83, p < 0.001). This study emphasizes the importance of robust patient selection criteria, especially for cases with borderline coronary anatomy.

Conclusion: Hemodynamic instability and moderate left main coronary artery stenosis significantly contribute to OPCAB-to-ONCAB conversion. Enhanced preoperative imaging and hemodynamic management protocols can potentially reduce conversion rates, improving surgical outcomes.

## Introduction

Coronary artery disease (CAD) is a leading global cause of morbidity and mortality, accounting for approximately nine million deaths annually. It is characterized by the buildup of atherosclerotic plaques within the coronary arteries, impairing blood and oxygen supply to the myocardium. Ischemic heart disease, a major manifestation of CAD, is the most prevalent cardiovascular illness worldwide and presents a significant challenge to sustainable global healthcare systems in the 21st century [1].

Coronary artery bypass grafting (CABG) is a widely established surgical revascularization technique designed to restore myocardial perfusion, significantly enhancing both survival and quality of life for patients with advanced CAD [2]. Traditionally, CABG is performed using cardiopulmonary bypass (CPB), which requires temporarily stopping the heart and maintaining circulation through an external heart-lung machine. However, CPB is associated with potential complications, including systemic inflammatory response, renal dysfunction, and neurocognitive impairment, particularly in elderly patients or those with multiple comorbidities [3].

To minimize the risks associated with CPB, off-pump coronary artery bypass (OPCAB) was developed. Unlike traditional CABG, OPCAB is performed on a beating heart without the need for CPB, offering several clinical advantages. These include reduced systemic inflammation, a lower risk of acute kidney injury, shorter ICU stays, and faster recovery [4]. Despite these benefits, technical challenges can sometimes necessitate conversion to on-pump coronary artery bypass (ONCAB), a shift associated with higher morbidity and potentially adverse outcomes. The conversion rate varies widely across institutions, influenced by factors like coronary anatomy, institutional protocols, and surgical experience. Identifying factors predictive of OPCAB-to-ONCAB conversion is essential for optimizing patient selection and enhancing surgical planning.

In our high-volume center, certain patients undergo OPCAB despite challenging coronary targets. Comorbidities such as renal dysfunction or bleeding risks often influence this decision, making OPCAB the preferred approach when possible. However, in cases with complex coronary anatomy, the possibility of conversion remains. This study aims to identify predictors and causes for intra-operative conversion to ONCAB, providing insights into both anatomical and procedural factors that can guide preoperative decision-making.

Conversion from OPCAB to ONCAB, although relatively uncommon, is associated with worse outcomes compared to patients who complete OPCAB without conversion. Reports suggest that conversion rates vary between 2.6% and 12%, with outcomes influenced by factors such as surgical team experience, patient selection, and institutional practices [5,6]. Patients who undergo conversion are more likely to experience higher morbidity, longer hospital stays, and increased short- and long-term mortality [7]. Hemodynamic instability, one of the most common reasons for conversion, has been linked with severe postoperative complications such as stroke and myocardial infarction [8].

Several studies have attempted to identify reliable predictors of conversion, including significant left main coronary artery stenosis, poor left ventricular function, and the extent of coronary artery disease [9]. However, variability in findings across studies suggests the need for further investigation to establish consistent predictors that can guide surgical planning.

This retrospective study aims to assess the incidence of intraoperative conversion from OPCAB to ONCAB and to identify key preoperative and intraoperative predictors of conversion at a high-volume cardiac center. By gaining deeper insights into the factors associated with conversion, this study seeks to refine patient selection criteria, enhance surgical planning, and ultimately improve patient outcomes by minimizing the need for conversion.

## Materials and methods

Study design

This retrospective case-control study was conducted to identify factors associated with intraoperative conversion from OPCAB to ONCAB. A retrospective design was chosen to utilize historical clinical data, enabling efficient evaluation of outcomes over a defined period. This approach is well-suited for studying rare events like OPCAB-to-ONCAB conversion.

Setting

The study was conducted at the Peshawar Institute of Cardiology, a high-volume tertiary care center in Peshawar, Pakistan, known for standardized surgical protocols and consistent clinical practices. Data were extracted from the institute’s electronic health records (EHR) system to ensure comprehensive and high-quality information for analysis.

Patient flow and participant selection

From an initial cohort of 760 patients screened for OPCAB eligibility, 46 patients (6.1%) were excluded due to incomplete medical records or concurrent procedures such as valve replacements. The final cohort consisted of 714 patients who underwent isolated OPCAB. Among these, 27 patients (3.78%) required intraoperative conversion to ONCAB. To maintain temporal matching and ensure comparability, 108 controls (15.1%) who successfully completed OPCAB without conversion were randomly selected. Controls were matched by the date of surgery to account for potential confounding due to changes in surgical practices over time.

Surgical procedures

All surgeries were performed by a single experienced surgical team following standardized protocols. Off-pump coronary artery bypass grafting was conducted through a median sternotomy with the use of the left internal mammary artery (LIMA) and saphenous vein grafts for coronary anastomosis. Coronary vessel immobilization was achieved using the Octopus stabilization system (Medtronic, Minneapolis, MN), and intracoronary shunts were employed to maintain myocardial perfusion during grafting.

In cases of intraoperative conversion to ONCAB, standard CPB techniques were employed. These included aorto-right atrial cannulation, application of an aortic cross-clamp, and administration of cardioplegia to induce cardiac arrest. Anticoagulation was reversed using protamine sulfate at the end of the procedure to ensure hemostasis before cannula removal.

Intraoperative conversion criteria and protocols

Conversion to ONCAB was considered in cases of hemodynamic instability, poor-quality target vessels, inadequate visualization, or technical challenges during coronary anastomosis. Specific intraoperative protocols were in place to prevent conversion, including the use of inotropic agents, intra-aortic balloon pump insertion, and strategic repositioning of the heart.

Patients with "poor-quality" coronary targets were defined based on vessel size, location, and calcification. Vessels with diameters less than 1.5 mm or extensive calcification were classified as high-risk for OPCAB. In cases where OPCAB was attempted despite these challenges, it was due to comorbid conditions that contraindicated CPB. For these challenging vessels, intracoronary shunts between 1.5 and 2.5 mm were used to maintain perfusion, while needles with a 0.8 mm diameter were standard for delicate anastomoses. Specific positioning techniques, including controlled heart manipulation and retraction adjustments, were used to improve access. Conversion to ONCAB was typically considered if challenges persisted after the LIMA grafting or during lateral wall grafting stages.

Data collection

Demographic details (age, gender, BMI), clinical history (e.g., diabetes, hypertension), and preoperative findings (ejection fraction, angiographic results) were extracted from the EHRs. The degree of left main coronary artery stenosis was categorized as none, 50%-70%, or >70%. Data were independently reviewed and cross-verified by two researchers to ensure accuracy and completeness.

Statistical analysis

Data were analyzed using STATA version 12.0 (StataCorp LLC, College Station, TX). Continuous variables were expressed as means with standard deviations (SD), while categorical variables were presented as frequencies and percentages. Comparisons between groups were made using t-tests for continuous variables and chi-square tests for categorical variables.

Multivariate logistic regression was performed to identify independent predictors of conversion. Variables with a p-value ≤0.25 in univariate analysis were included in the model. Statistical significance was defined as p <0.05.

Ethical considerations

This study was approved by the Institutional Review Board (IRB) of the Peshawar Institute of Cardiology, Peshawar, Pakistan, on December 8, 2023 (approval no. IRC/24/63). A waiver of informed consent was granted due to the retrospective nature of the study. The study adhered to the ethical standards outlined in the Declaration of Helsinki.

## Results

A total of 714 patients underwent isolated OPCAB at the Peshawar Institute of Cardiology. Of these, 27 (3.78%) required conversion to ONCAB, while 687 (96.2%) successfully completed OPCAB without conversion. For analysis, the 27 conversion cases were compared with 108 randomly selected controls (15.1%) who underwent OPCAB without conversion.

The baseline characteristics of the participants are summarized in Table 1. The mean age was 59.0 ± 8.77 years, with no statistically significant difference between the conversion and non-conversion groups (59.56 ± 7.81 vs. 58.8 ± 8.97 years; p = 0.68). Male participants made up 82.96% of the sample, with a similar distribution across the two groups (p = 0.73). The conversion group had a slightly higher mean body mass index (BMI) of 28.30 ± 3.55 kg/m² compared to the controls (27.19 ± 4.19 kg/m²), although this difference was not statistically significant (p = 0.19).

**Table 1:** Baseline characteristics of patients undergoing OPCAB and conversion to ONCAB Values are presented as means with standard deviations (SD) for continuous variables and as N (%) for categorical variables. A p-value <0.05 indicates a statistically significant difference. OPCAB: off-pump coronary artery bypass; ONCAB: on-pump coronary artery bypass; BMI: body mass index

Characteristic	Conversion to ONCAB (N = 27)	OPCAB without conversion (N = 108)	p-value
Age (years, mean ± SD)	59.56 ± 7.81	58.8 ± 8.97	0.68
Male sex, n (%)	23 (85.19)	89 (82.40)	0.73
BMI (kg/m², mean ± SD)	28.30 ± 3.55	27.19 ± 4.19	0.19
Hypertension, n (%)	18 (66.67)	54 (50.00)	0.13
Diabetes mellitus, n (%)	12 (44.44)	43 (39.81)	0.66
Current smoker, n (%)	12 (44.44)	41 (37.96)	0.54

Preoperative cardiac and angiographic findings are detailed in Table 2. A left ventricular ejection fraction (LVEF) of ≤40% was observed in seven (25.93%) of the conversion cases compared to 20 (18.52%) of the controls, although this difference was not statistically significant (p = 0.16). Left main coronary artery stenosis of 50%-70% was significantly associated with conversion, with 15 (55.56%) of the cases exhibiting this degree of stenosis compared to 13 (12.04%) of the controls (p = 0.0004). All patients with >70% stenosis were managed with ONCAB directly and were not included in the conversion cohort. The extent of CAD, indicated by triple-vessel involvement, was similar between the groups (21 (77.78%) in the cases vs. 75 (69.45%) in the controls, p = 0.49).

**Table 2:** Preoperative cardiac and angiographic findings LVEF: left ventricular ejection fraction; ONCAB: on-pump coronary artery bypass grafting; OPCAB: off-pump coronary artery bypass grafting; n: number of patients; SD: standard deviation; p-value: probability value (significant if less than 0.05).

Characteristic	Conversion to ONCAB (N=27)	OPCAB without conversion (N=108)	p-value
LVEF ≤40%, n (%)	7 (25.93)	20 (18.52)	0.16
Left main stenosis, n (%)			
- None	12 (44.44)	79 (73.15)	0.0004
- 50%-70%	15 (55.56)	13 (12.04)	0.0004
- >70%	0 (0.00)	16 (14.81)	-
Triple-vessel disease, n (%)	21 (77.78)	75 (69.45)	0.49

The primary reasons for intraoperative conversion to ONCAB are outlined in Table 3. Hemodynamic instability was the most common cause, accounting for 18 (66.67%) of the conversions. Within this group, 15 (83.33%) experienced persistent hypotension, while three (16.67%) had arrhythmias. Poor target vessel quality contributed to four (14.81%) of the conversions, followed by inadequate visualization in two (7.41%) cases and the need for adjunctive procedures such as endarterectomy or long patch plasty in three (11.11%) cases.

**Table 3:** Reasons for intraoperative conversion to ONCAB ONCAB: on-pump coronary artery bypass grafting; N: number of patients; %: percentage of total conversions

Reason for conversion to ONCAB	N (%)
Hemodynamic instability	18 (66.67)
- Persistent hypotension	15 (83.33)
- Arrhythmias	3 (16.67)
Poor target vessel quality	4 (14.81)
Inadequate visualization	2 (7.41)
Endarterectomy/long patch plasty	3 (11.11)

Persistent hemodynamic instability was observed in 66.67% of cases, making it the most frequent cause of conversion. During these episodes, several key metrics were monitored. End-tidal carbon dioxide (CO₂) fluctuations and ST-segment changes provided indicators of perfusion status, while a transit flow meter assessed conduit flow through grafts. The average duration of instability before conversion was approximately 15 minutes, with a range from 10-20 minutes. Inotropic support was provided as a first-line response, followed by repositioning and adjustment of heart manipulation techniques.

Conversion was most likely during lateral wall grafting, typically after the LIMA graft was completed. The technical challenges of accessing lateral vessels often necessitated conversion, particularly in patients with borderline hemodynamics. Detailed procedural data indicate that 15 of the 27 conversions occurred at this stage, emphasizing the added risk associated with challenging anatomical locations.

Postoperative outcomes are summarized in Table 4. Procedural success was achieved in 25 (92.6%) of the conversion cases and 105 (97.2%) of the controls, although the difference was not statistically significant (p = 0.092). The incidence of stent thrombosis and restenosis was also comparable between the two groups, with no statistically significant differences.

**Table 4:** Postoperative outcomes n: number of patients; %: percentage; ONCAB: on-pump coronary artery bypass grafting; OPCAB: off-pump coronary artery bypass grafting; p-value: probability value (significant if less than 0.05).

Outcome	Conversion to ONCAB (n=27)	OPCAB without conversion (n=108)	p-value
Procedural success	25 (92.6%)	105 (97.2%)	0.092
Stent thrombosis	1 (3.7%)	2 (1.9%)	0.354
Restenosis	2 (7.4%)	4 (3.7%)	0.301

Multivariate logistic regression identified left main coronary artery stenosis of 50%-70% as the only independent predictor of conversion, with an odds ratio (OR) of 7.60 (95% CI: 2.91-19.83, p = 0.0001). None of the other variables, including age, sex, BMI, hypertension, diabetes, smoking status, or LVEF, were significantly associated with conversion risk, as shown in Table 5.

**Table 5:** Multivariate logistic regression analysis for predictors of intraoperative conversion to ONCAB This table displays the results of the logistic regression analysis, identifying significant predictors of conversion from OPCAB to ONCAB. The OR with 95% CI and p-values are reported. OPCAB: off-pump coronary artery bypass; ONCAB: on-pump coronary artery bypass; LVEF: left ventricular ejection fraction; BMI: body mass index

Variable	Odds ratio (OR)	95% Confidence interval (CI)	p-value
Age (years)	1.01	0.96 – 1.06	0.70
Male sex	1.23	0.38 – 3.96	0.73
BMI (kg/m²)	1.10	0.92 – 1.32	0.19
Hypertension	1.52	0.68 – 3.37	0.13
Diabetes mellitus	1.21	0.52 – 2.83	0.66
Smoking status	1.31	0.56 – 3.07	0.54
LVEF ≤40%	1.45	0.58 – 3.64	0.16
Left main stenosis (50-70%)	7.60	2.91 – 19.83	0.0001
Triple-vessel disease	1.25	0.55 – 2.85	0.49

The overall incidence of intraoperative conversion from OPCAB to ONCAB was 3.78%, with hemodynamic instability being the most frequent cause of conversion. Left main stenosis of 50%-70% was identified as the sole independent predictor of conversion, highlighting the need for thorough preoperative evaluation in patients with moderate left main stenosis to reduce the risk of conversion and optimize surgical outcomes.

## Discussion

Off-pump coronary artery bypass grafting offers several advantages over conventional ONCAB, including reduced systemic inflammation, shorter ICU stays, decreased operative trauma, and faster rehabilitation. These benefits make OPCAB particularly advantageous for selected patients, especially those at higher risk for complications associated with CPB [4]. However, the success of OPCAB largely depends on patient selection and surgical expertise, as technical challenges or hemodynamic instability may necessitate intra-operative conversion to ONCAB [5].

In this study, the conversion rate from OPCAB to ONCAB was 3.78%, consistent with the 3.1% conversion rate reported by Chakravarthy et al. [6]. However, conversion rates vary across studies, with Borde et al. reporting a 6.49% rate and Légaré et al. documenting rates ranging from 5% to 8% [7, 9]. These discrepancies highlight the role of institutional protocols, patient selection, and surgical team experience in shaping outcomes, underscoring the importance of refining these factors to minimize conversions.

Our findings show that hemodynamic instability was the most common reason for conversion, occurring in 66.67% of cases, which aligns with previous studies [7]. Persistent hypotension and ST-segment changes were primary indicators before conversion, highlighting the importance of continuous intraoperative monitoring. Similarly, Edgerton et al. identified inadequate myocardial perfusion during OPCAB as a major contributor to conversion, advocating for preemptive strategies such as inotropic support and intra-aortic balloon pumps to prevent hemodynamic collapse [8].

The analysis also identified left main coronary artery stenosis of 50%-70% as the only independent predictor of conversion (OR: 7.60, 95% CI: 2.91-19.83, p < 0.001). This finding aligns with the work of Gan et al., who reported an elevated risk of conversion among patients with moderate left main stenosis due to increased hemodynamic instability during OPCAB [10]. In our cohort, patients with >70% stenosis were directly managed with ONCAB, reinforcing the view that severe stenosis serves as a contraindication for OPCAB. This emphasizes the need for robust patient selection criteria, especially for cases with borderline coronary anatomy.

Interestingly, Chen et al. found no significant relationship between left main stenosis and conversion rates, which may reflect advancements in surgical tools and stabilizing devices, as well as increasing expertise among surgical teams [11]. Similarly, Takagi et al. suggested that with growing proficiency in off-pump techniques, anatomical challenges like left main stenosis become less impactful on conversion outcomes [5]. These contrasting findings highlight the evolving nature of surgical practice and the importance of continuous training and adaptation to new technologies.

We found no significant associations between conversion and demographic or clinical factors such as age, gender, BMI, hypertension, diabetes, or smoking status. This aligns with the results of Yoon et al., who also reported no significant associations between these variables and conversion rates [12]. However, other studies suggest that advanced age and poor left ventricular function may indirectly increase conversion risk by complicating the operative course [11]. This indicates that while anatomical factors remain the primary predictors of conversion, clinical variables may influence outcomes by increasing procedural complexity.

Institutional protocols and surgical expertise play a crucial role in reducing conversion rates and improving outcomes. Keeling et al. demonstrated that conversions, whether planned or unplanned, are associated with increased 30-day mortality and complications, including renal failure and prolonged mechanical ventilation [13]. Our findings underscore the need for robust institutional protocols to prevent conversions and mitigate their associated risks. Maintaining coordination between surgical and anesthetic teams is essential to detecting early signs of ischemia and promptly addressing intraoperative hemodynamic instability.

Our study reinforces the importance of preoperative imaging and planning to identify high-risk patients, particularly those with borderline hemodynamics or complex coronary artery disease. Enhanced imaging technologies improve patient selection and surgical outcomes by minimizing intra-operative challenges.

Similarly, the research by Wang et al. underscores the critical role of surgical expertise, demonstrating that centers with extensive experience in OPCAB achieve better outcomes over time. They found that institutions with surgeons who performed more than 200 OPCAB procedures reported significantly lower conversion rates and better prognosis, echoing our findings that surgical experience reduces complications and enhances outcomes [14]. These studies confirm that both preoperative imaging and advanced intraoperative tools, such as 3D visualization, play pivotal roles in preventing conversion and optimizing surgical planning.

Looking ahead, future research should explore how emerging technologies, such as robotically assisted surgery and real-time imaging, can further reduce the need for conversion by enhancing surgical precision. Technological advancements hold significant potential for improving patient outcomes and advancing the field of minimally invasive cardiac surgery.

Limitations

The primary limitation of this study is its retrospective design, which may introduce selection bias and limit the ability to establish causality. Additionally, the relatively small number of conversion events reduces the statistical power to detect subtle differences between subgroups. Future research should focus on prospective, multi-center studies to validate these findings and identify additional predictors of conversion.

## Conclusions

This study identified that intraoperative conversion from OPCAB to ONCAB occurred in 3.78% of cases, primarily due to hemodynamic instability, with left main coronary artery stenosis of 50%-70% emerging as the only significant predictor. These findings underscore the importance of thorough preoperative evaluation and planning for patients with moderate left main stenosis to optimize surgical decision-making and minimize conversion risks. Incorporating these insights into patient selection and preparation could enhance outcomes in CABG. Future research should focus on prospective studies and multi-center comparisons to validate these findings and further refine risk assessment strategies.
